# Supplementary material for: Molecular EPISTOP, a comprehensive multi-omic analysis of blood from Tuberous Sclerosis Complex infants age birth to two years
Source: Nat Commun. 2023 Nov 23;14:7664. doi: 10.1038/s41467-023-42855-6 (PMC10667269; doi:10.1038/s41467-023-42855-6)
Supplement: Supplementary file 1 — Supplementary Information [file 41467_2023_42855_MOESM1_ESM.pdf]

TITLE:

Molecular EPISTOP, a comprehensive multi-omic analysis of blood from Tuberous Sclerosis Complex infants age birth to two years

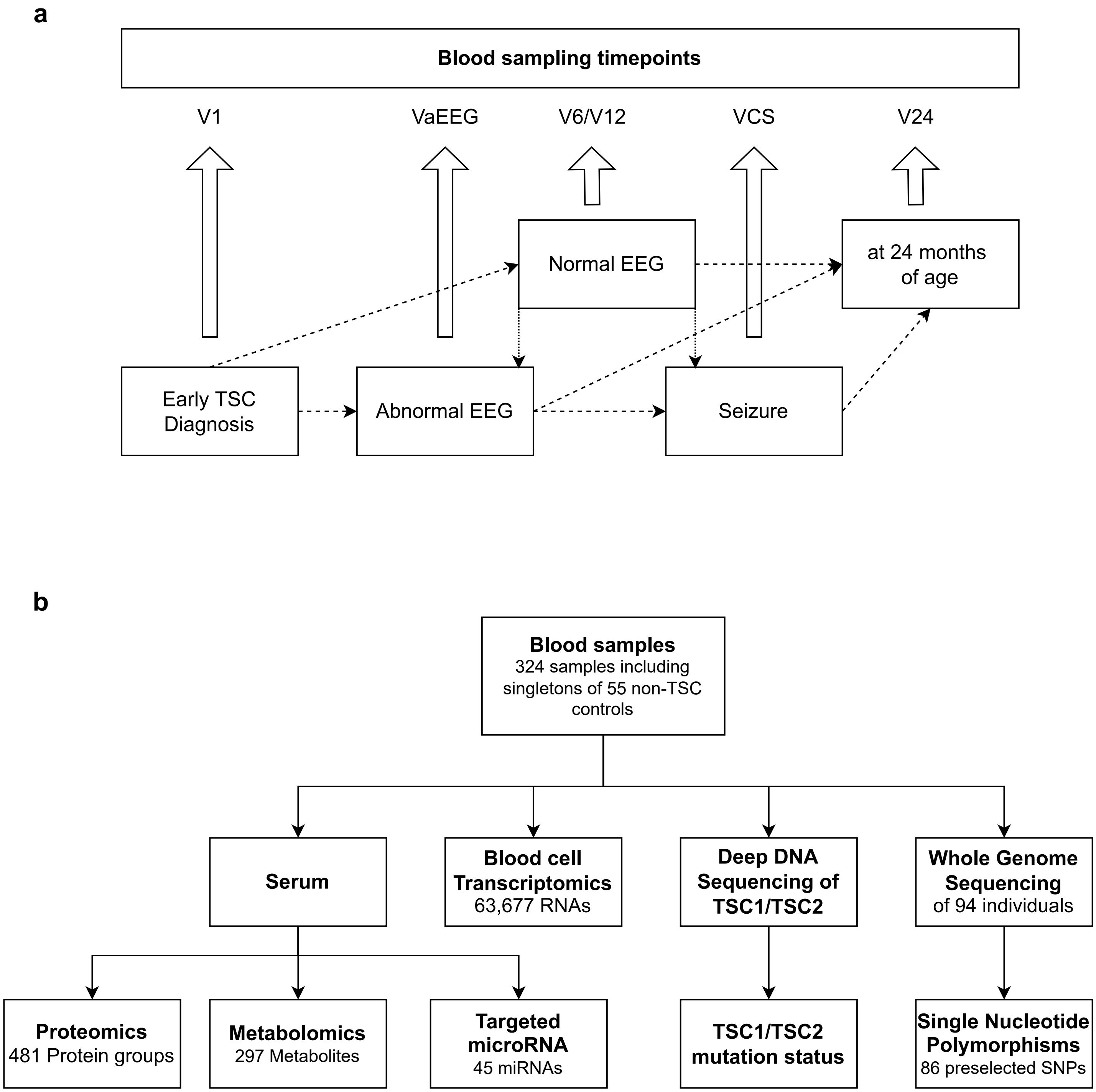
a
Blood sampling timepoints
V1
VaEEG
V6/V12
VCS
V24
Normal EEG
at 24 months of age
Early TSC Diagnosis
Abnormal EEG
Seizure
b
Blood samples
324 samples including singletons of 55 non-TSC controls
Serum
Blood cell Transcriptomics
63,677 RNAs
Deep DNA Sequencing of TSC1/TSC2
Whole Genome Sequencing
of 94 individuals
Proteomics
481 Protein groups
Metabolomics
297 Metabolites
Targeted microRNA
45 miRNAs
TSC1/TSC2 mutation status
Single Nucleotide Polymorphisms
86 preselected SNPs

Supplementary Figure 1. Blood sampling timepoints (a), and sample material (b) in the EPISTOP project. V1 blood sample is collected at study entry (age birth to 4 months); VaEEG blood sample taken after abnormal electroencephalogram (EEG); V6/V12 blood sample taken at age 6 or 12 months, respectively, if no abnormal EEG or seizure onset has occurred; VCS blood sample is taken after onset of epilepsy (also subclinical seizure detected via EEG); V24 blood sample taken at age 24 months in all subjects.

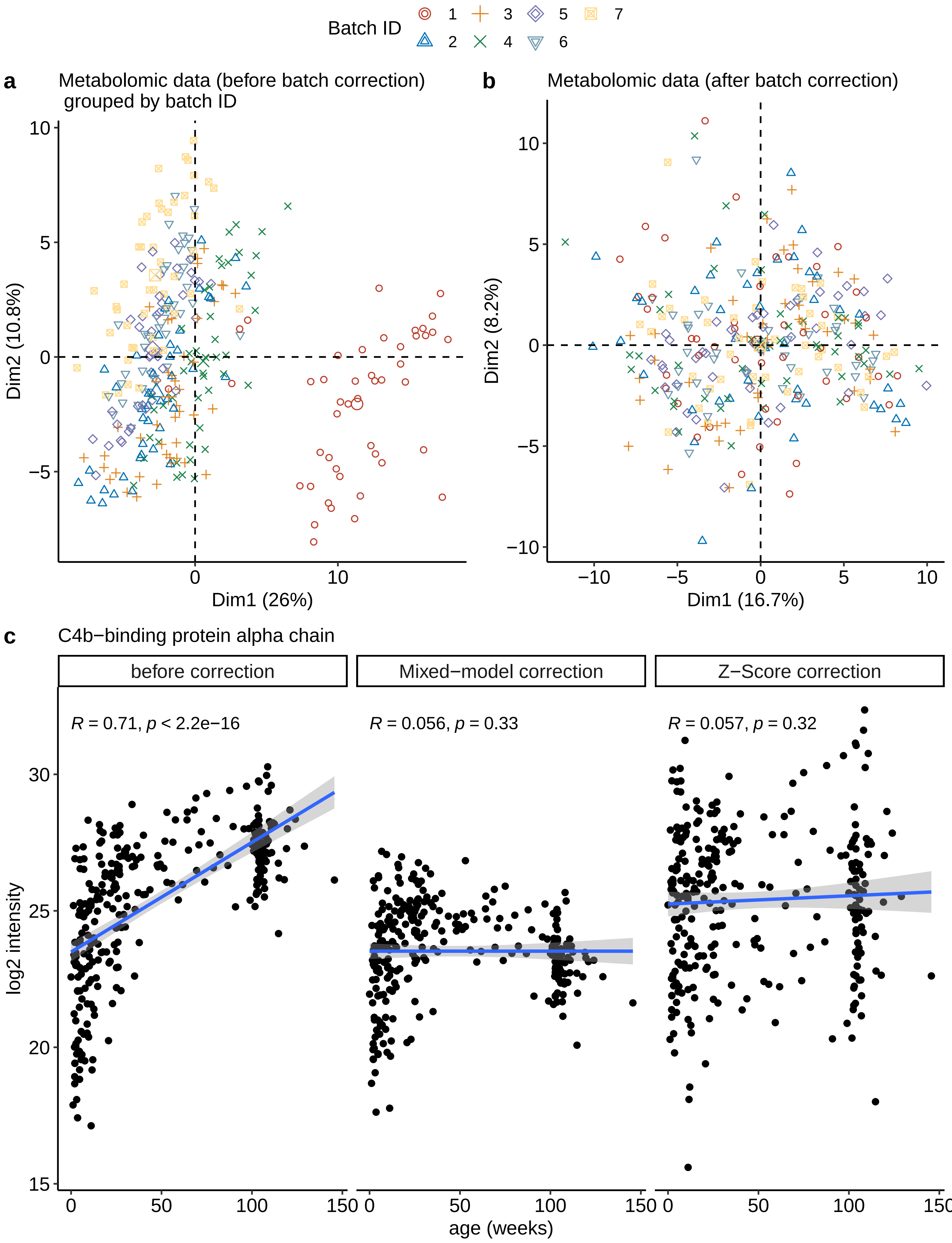
Batch ID
1
2
3
4
5
6
7
a
Metabolomic data (before batch correction)
grouped by batch ID
Dim1 (26%)
Dim2 (10.8%)
b
Metabolomic data (after batch correction)
Dim1 (16.7%)
Dim2 (8.2%)
c
C4b−binding protein alpha chain
before correction
Mixed−model correction
Z−Score correction
R = 0.71, p < 2.2e−16
R = 0.056, p = 0.33
R = 0.057, p = 0.32
log2 intensity
age (weeks)

Supplementary Figure 2. Examples of confounding effects before and after correction.

ab. In PCA of log2 intensities, colored by batch, observed batch effects in the metabolite data (a) was corrected by a Z-score un-Z-score method (b).

c. Developmental effects visible in all datasets were corrected by the same method or by a linear mixed model correction approach. Exemplary a scatter plot of log2 intensities of C4b-binding protein alpha chain according to age, before (c, left) and after correction by linear mixed models (LMM,c, middle) and Z-score method (c, right) is shown. Spearman's Rank correlation coefficients (R) are not significant after correction via either method. The line is drawn to visualize the trend in the data, representing the linear regression fit, while the surrounding shaded area denotes the 95% confidence intervals (CI). Source data are provided as a Source Data file.

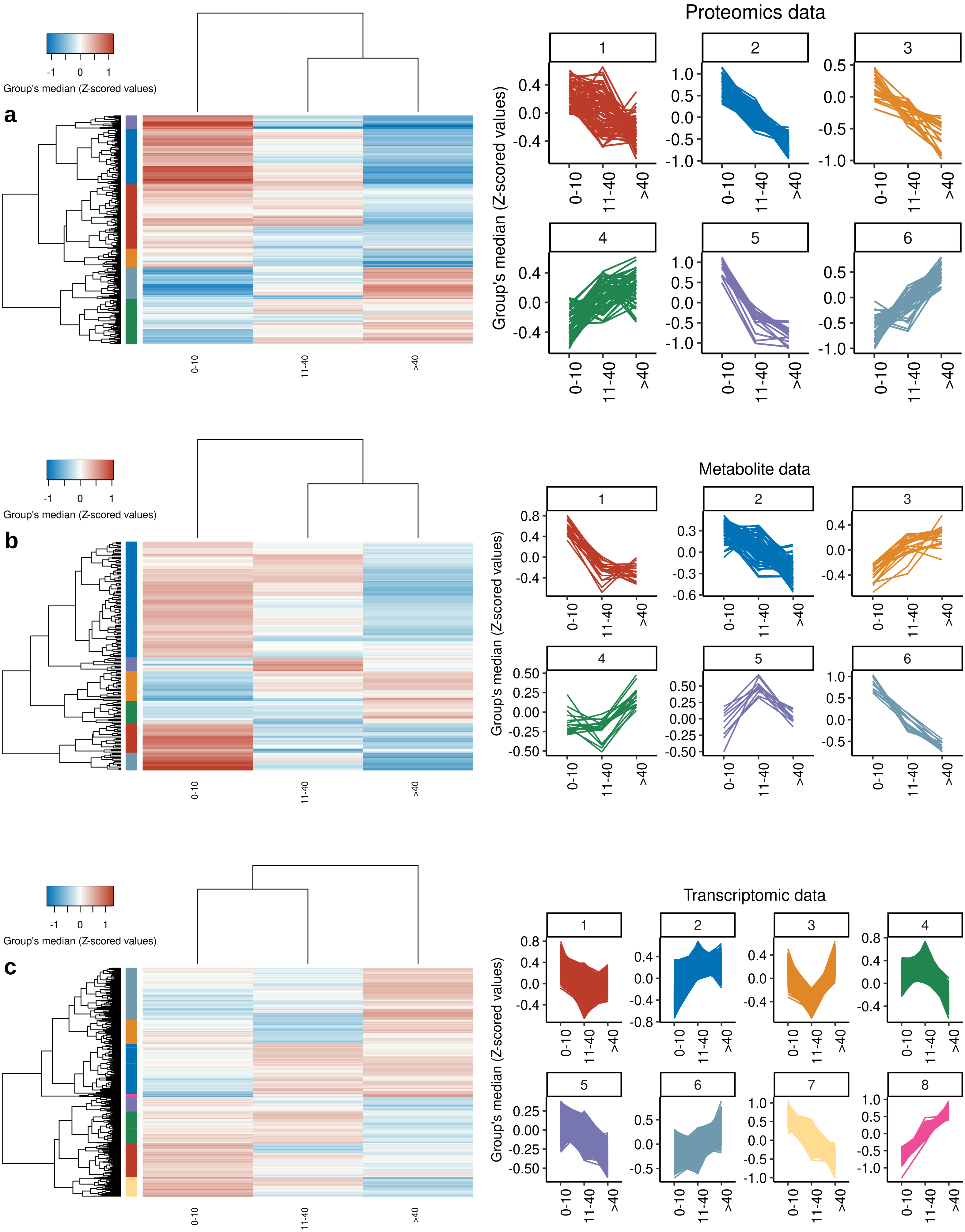

a
b
c
Group's median (Z-scored values)
Proteomics data
Metabolite data
Transcriptomic data
Group's median (Z-scored values)
0-10
11-40
>40

Supplementary Figure 3. Developmental changes in proteomics, metabolite, and transcriptomic data.

abc. Heatmap of hierarchically clustered group median Z-scores of analytes that were significantly different according to age by Kruskal-Wallis. 289 of 340 (85%) protein groups (a), 173 of 249 (69%) metabolites (b), and 10506 of 20579 (51%) RNAs (c) showed significant differences in comparing age groups 0-10 weeks, 11-40 weeks, and > 40 weeks (FDR < 0.05). Red color indicates high expression and blue indicates low expression using Z-scores. Analytes with similar patterns of change, were grouped into 6 or 8 clusters. Clusters are highlighted with different colors. Line plots for all analytes in each cluster are shown at right. Source data are provided as a Source Data file.

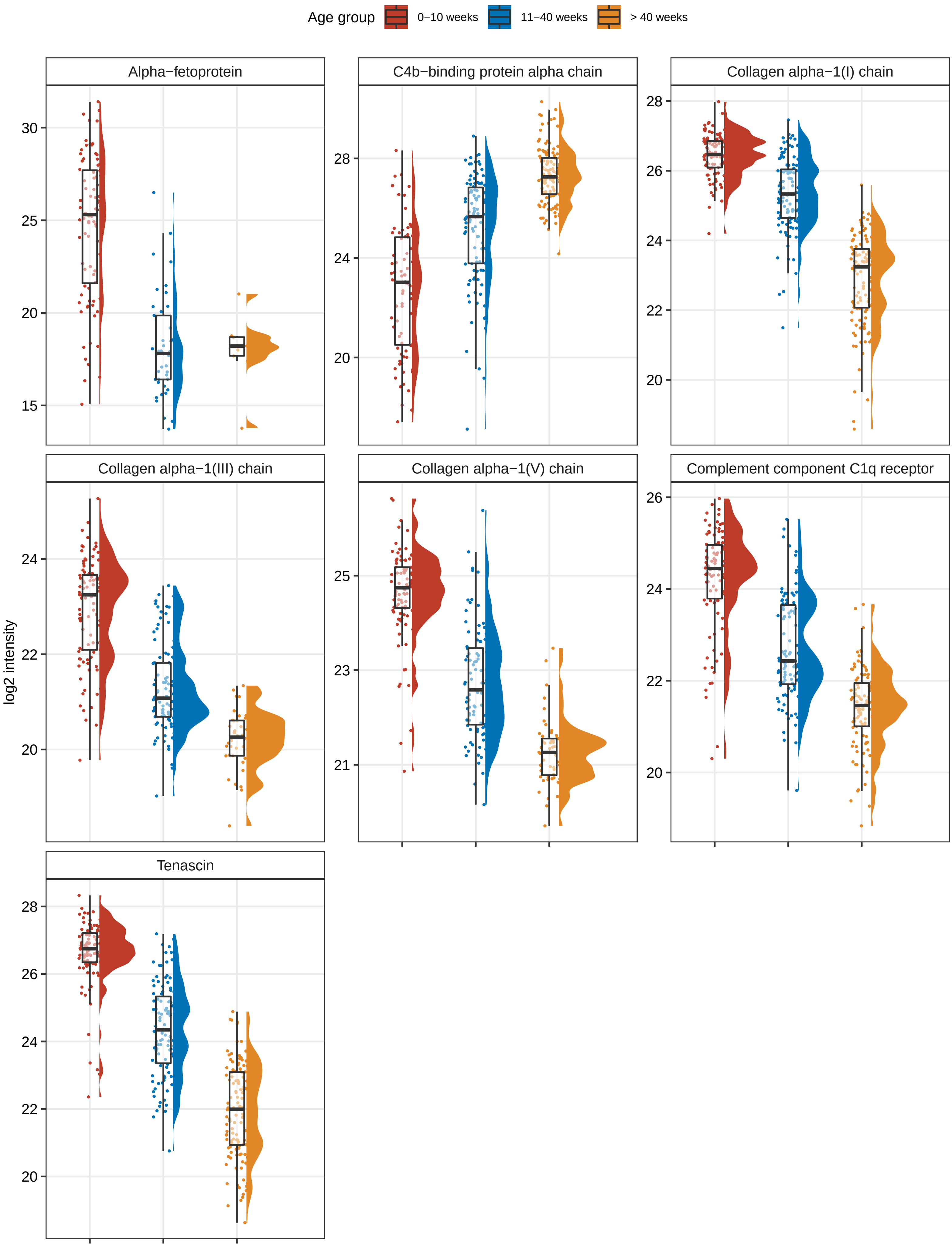

Age group
0−10 weeks
11−40 weeks
> 40 weeks
Alpha−fetoprotein
C4b−binding protein alpha chain
Collagen alpha−1(I) chain
Collagen alpha−1(III) chain
Collagen alpha−1(V) chain
Complement component C1q receptor
Tenascin
log2 intensity

Supplementary Figure 4. Examples of protein groups changing with age. In a three group comparison (0-10 weeks versus 11-40 weeks versus > 40 weeks of age) multiple protein groups exhibited median fold changes > 7 and were significant in Kruskal-Wallis analysis (FDR < 0.05). The box's middle line marks the median, its edges represent the 25th and 75th percentiles, and whiskers extend to data points within 1.5*IQR, with points beyond as outliers. The density plot illustrates the data’s distribution, highlighting areas of high and low frequencies to showcase the variability in the data values. Source data are provided as a Source Data file.

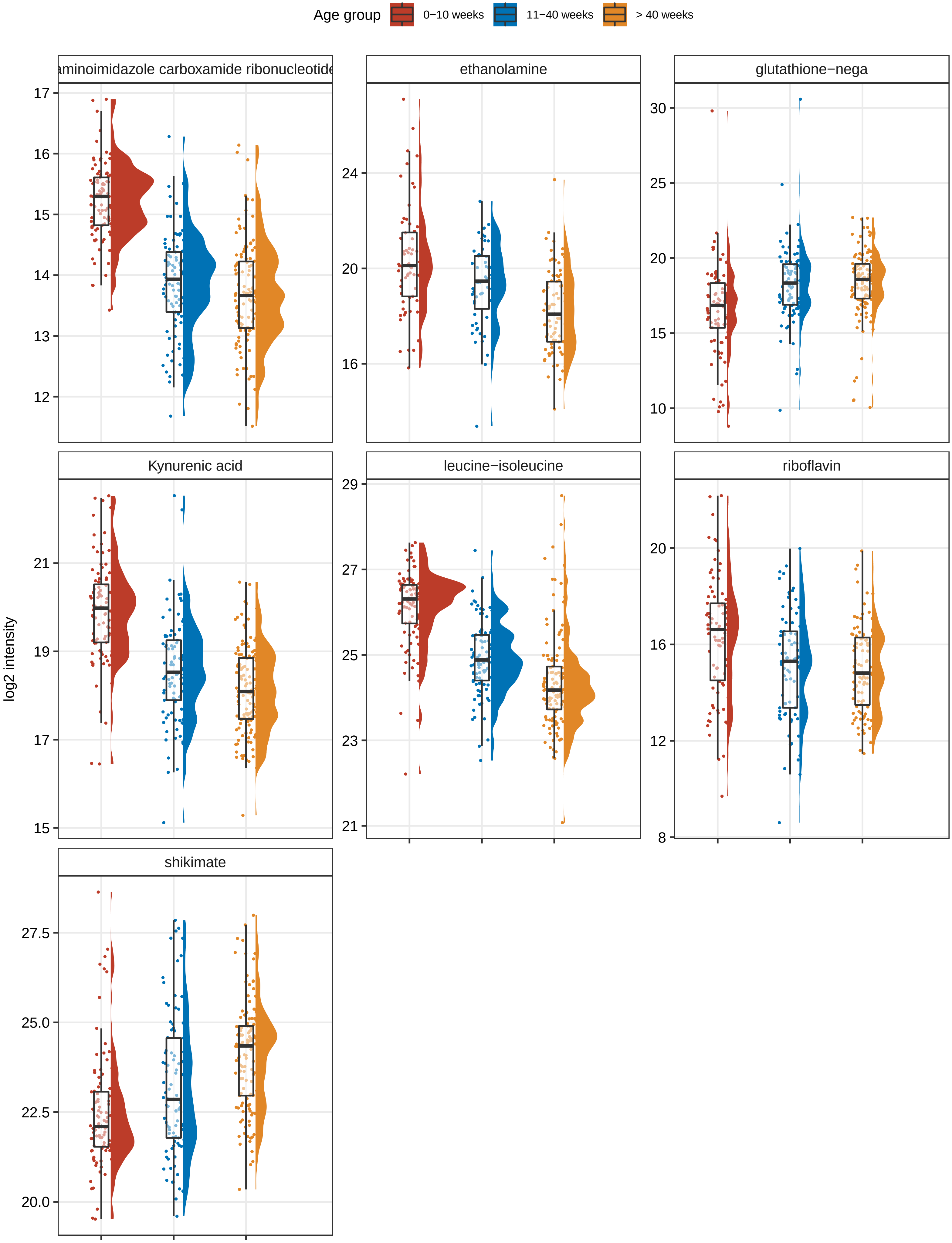
Age group
0−10 weeks
11−40 weeks
> 40 weeks
aminoimidazole carboxamide ribonucleotide
ethanolamine
glutathione−nega
Kynurenic acid
leucine−isoleucine
riboflavin
shikimate
log2 intensity
12
13
14
15
16
17
16
20
24
10
15
20
25
30
15
17
19
21
21
23
25
27
29
8
12
16
20
20.0
22.5
25.0
27.5

Supplementary Figure 5. Examples of metabolites changing with age. Three group comparison (0-10 weeks versus 11-40 weeks versus > 40 weeks of age) plots are shown for seven metabolites with the largest median fold change, > 3, which were also significant by Kruskal-Wallis analysis (FDR < 0.05). The box's middle line marks the median, its edges represent the 25th and 75th percentiles, and whiskers extend to data points within 1.5*IQR, with points beyond as outliers. The density plot illustrates the data’s distribution, highlighting areas of high and low frequencies to showcase the variability in the data values. Source data are provided as a Source Data file.

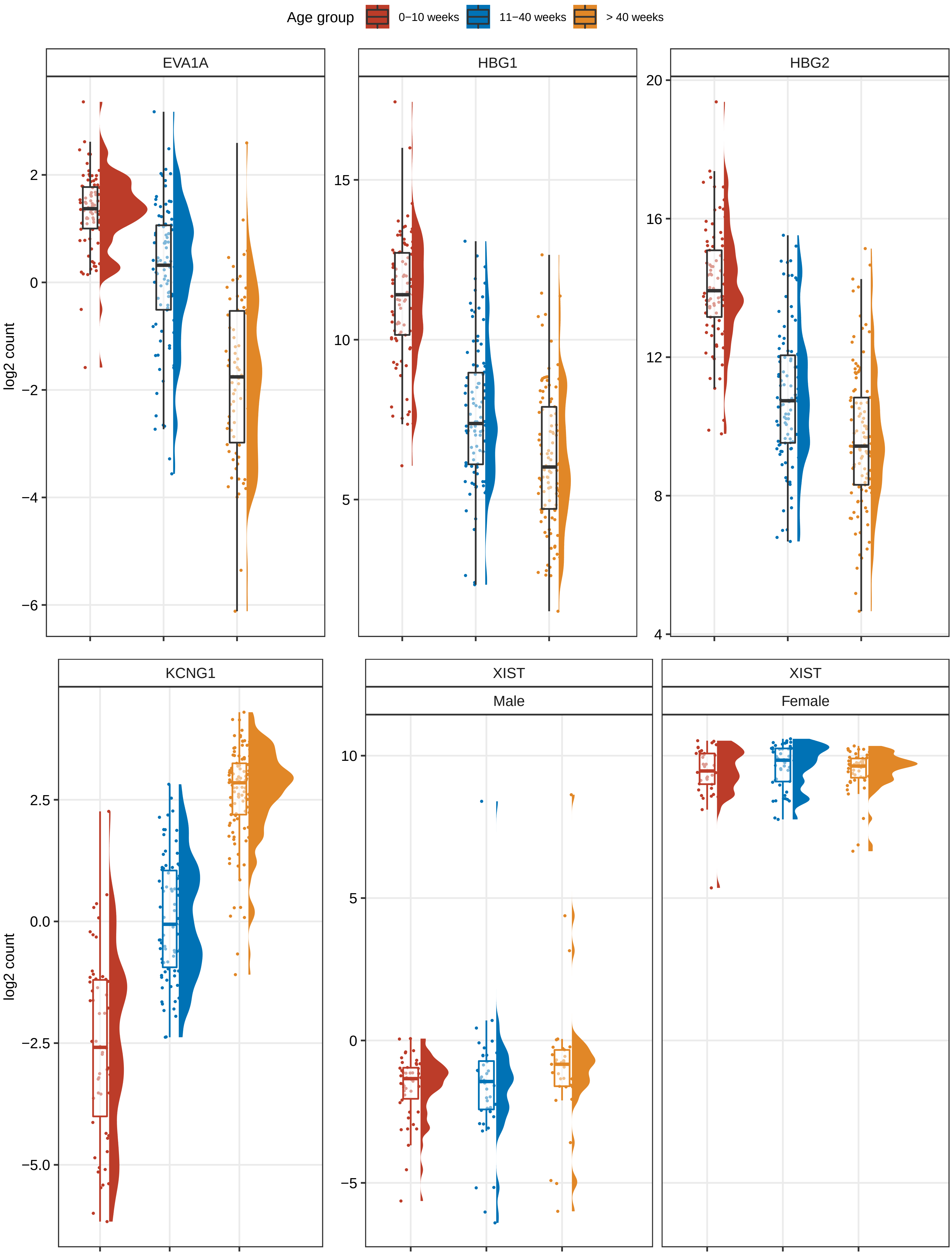
Age group
0–10 weeks
11–40 weeks
> 40 weeks
EVA1A
HBG1
HBG2
KCNG1
XIST
Male
XIST
Female
log2 count
log2 count

Supplementary Figure 6. Examples of RNAs changing with age, and *XIST*. In a three group comparison (0-10 weeks versus 11-40 weeks versus > 40 weeks of age) many genes showed significant differences (Kruskal-Wallis analysis (FDR < 0.05)) with median fold changes > 7. Four genes are shown here. *XIST* expression is also shown divided by age and sex. The box's middle line marks the median, its edges represent the 25th and 75th percentiles, and whiskers extend to data points within 1.5*IQR, with points beyond as outliers. The density plot illustrates the data's distribution, highlighting areas of high and low frequencies to showcase the variability in the data values. Source data are provided as a Source Data file.

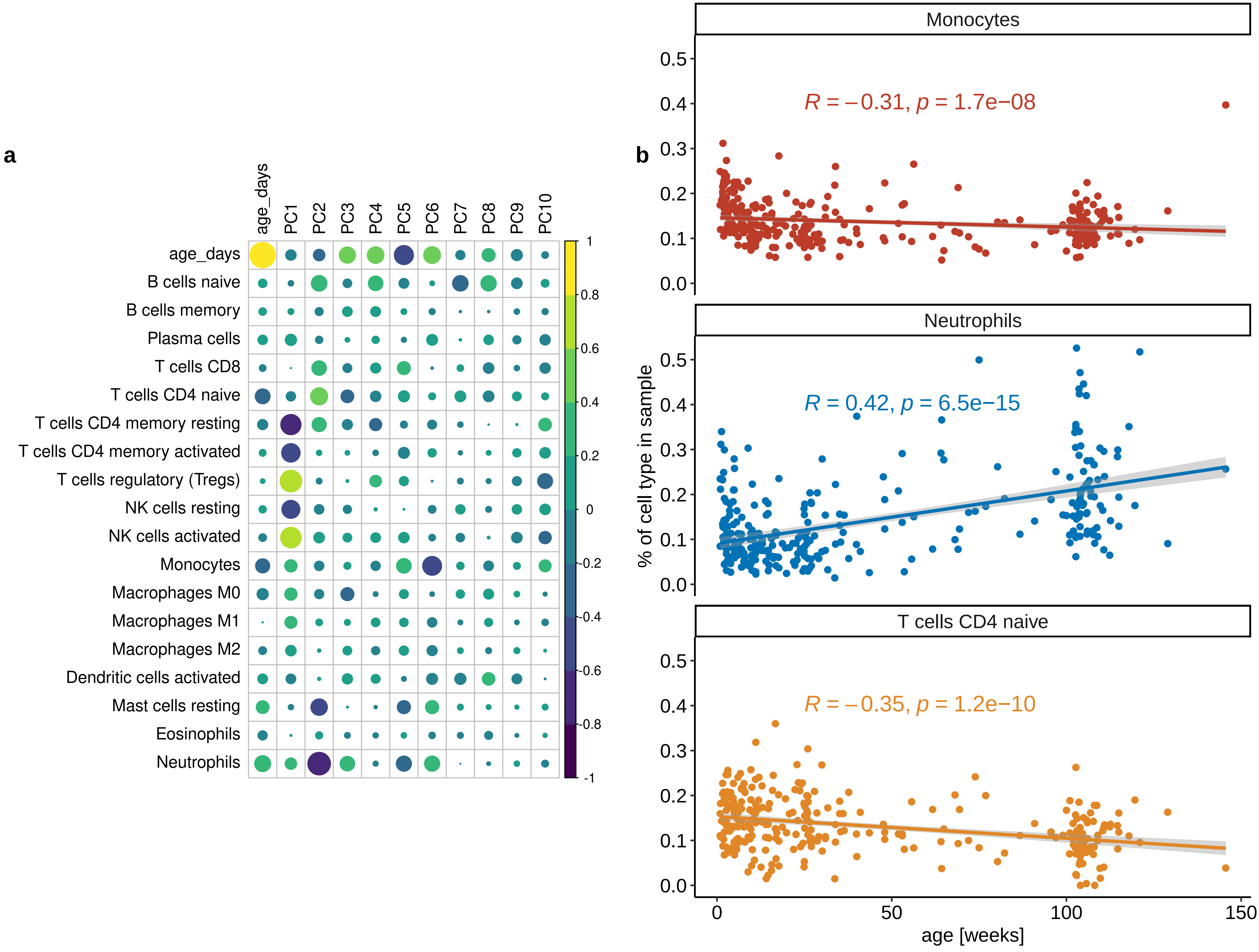
a
age_days
PC1
PC2
PC3
PC4
PC5
PC6
PC7
PC8
PC9
PC10
age_days
B cells naive
B cells memory
Plasma cells
T cells CD8
T cells CD4 naive
T cells CD4 memory resting
T cells CD4 memory activated
T cells regulatory (Tregs)
NK cells resting
NK cells activated
Monocytes
Macrophages M0
Macrophages M1
Macrophages M2
Dendritic cells activated
Mast cells resting
Eosinophils
Neutrophils
1
0.8
0.6
0.4
0.2
0
-0.2
-0.4
-0.6
-0.8
-1
b
Monocytes
R = −0.31, p = 1.7e−08
Neutrophils
R = 0.42, p = 6.5e−15
T cells CD4 naive
R = −0.35, p = 1.2e−10
% of cell type in sample
0.0
0.1
0.2
0.3
0.4
0.5
0
50
100
150
age [weeks]

Supplementary Figure 7. Cellular decomposition of gene expression data, and correlation with principal component analysis.

a. Correlation between the first 10 PCs, age, and cellular makeup of each sample as derived using CIBERSORT. The size and color of the nodes indicate the strength of the Spearman's Rank Coefficient between the two variables.

b. Scatter plots of percent of each cell type determined by CIBERSORT according to age in weeks, for monocytes, neutrophils, and naive CD4 T cells. The line is drawn to visualize the trend in the data, representing the linear regression fit, while the surrounding shaded area denotes the 95% confidence intervals (CI). Spearman's Rank correlation coefficients (R) and p values obtained by two-sided Spearman's correlation test are indicated.

Source data are provided as a Source Data file.

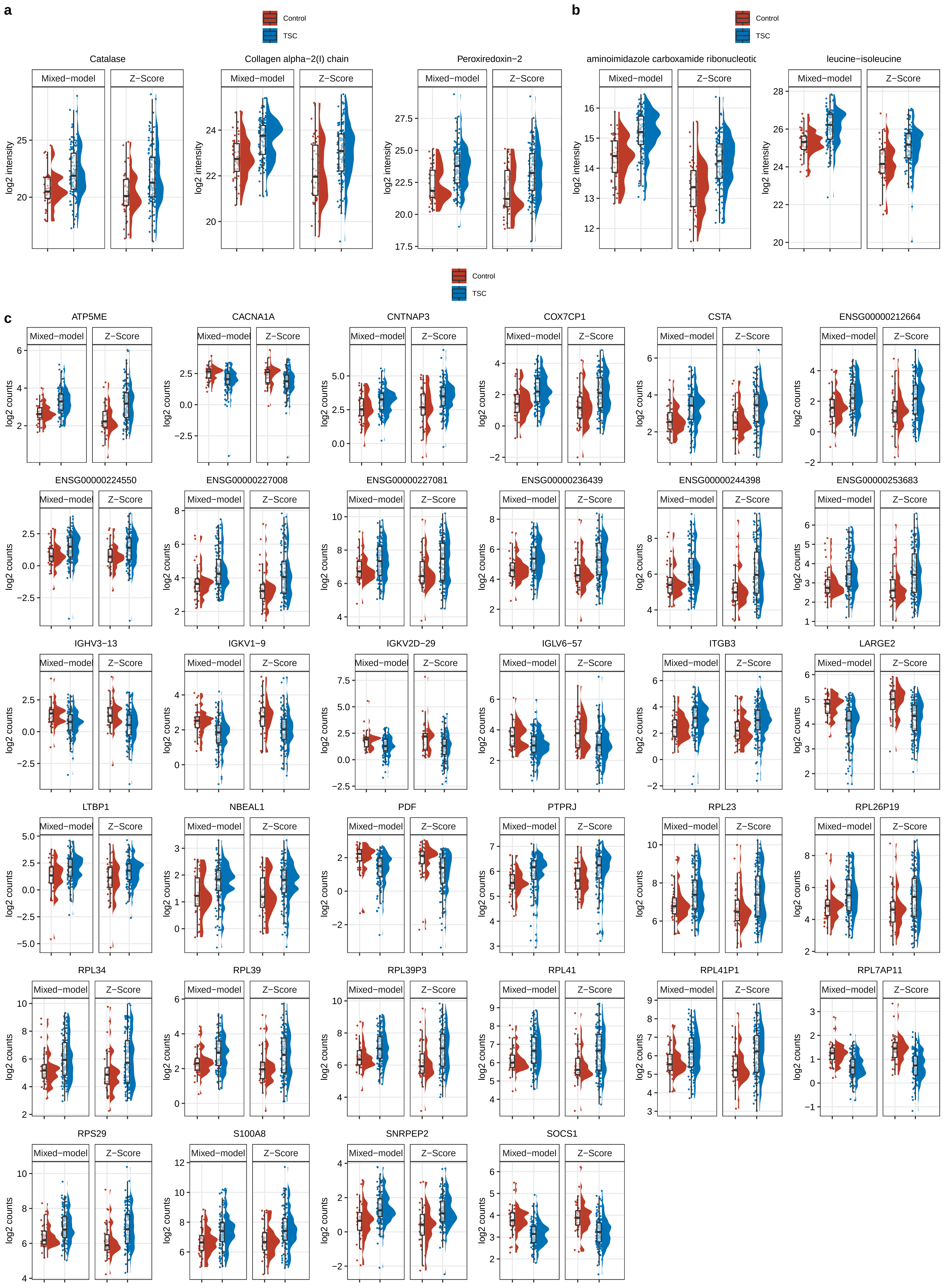

a
Control
TSC
Catalase
Collagen alpha−2(I) chain
Peroxiredoxin−2
Mixed−model
Z−Score
log2 intensity
b
Control
TSC
aminoimidazole carboxamide ribonucleotic
leucine−isoleucine
Mixed−model
Z−Score
log2 intensity
c
Control
TSC
ATP5ME
CACNA1A
CNTNAP3
COX7CP1
CSTA
ENSG00000212664
ENSG00000224550
ENSG00000227008
ENSG00000227081
ENSG00000236439
ENSG00000244398
ENSG00000253683
IGHV3−13
IGKV1−9
IGKV2D−29
IGLV6−57
ITGB3
LARGE2
LTBP1
NBEAL1
PDF
PTPRJ
RPL23
RPL26P19
RPL34
RPL39
RPL39P3
RPL41
RPL41P1
RPL7AP11
RPS29
S100A8
SNRPEP2
SOCS1
Mixed−model
Z−Score
log2 counts

Supplementary Figure 8. Analytes identified as significantly different between non-TSC controls and TSC subjects. a, Proteins; b, metabolites; c, RNA species with significant differences in a comparison of samples from individuals with TSC (without prior treatment with VGB and no previous seizure history) and non-TSC controls (FDR < 0.05; fold change > 1.5). The TSC group contains samples drawn at 0-24 months of age as well as samples from individuals that showed abnormal EEGs at sample draw. The box's middle line marks the median, its edges represent the 25th and 75th percentiles, and whiskers extend to data points within 1.5*IQR, with points beyond as outliers. The density plot illustrates the data's distribution, highlighting areas of high and low frequencies to showcase the variability in the data values. Source data are provided as a Source Data file.

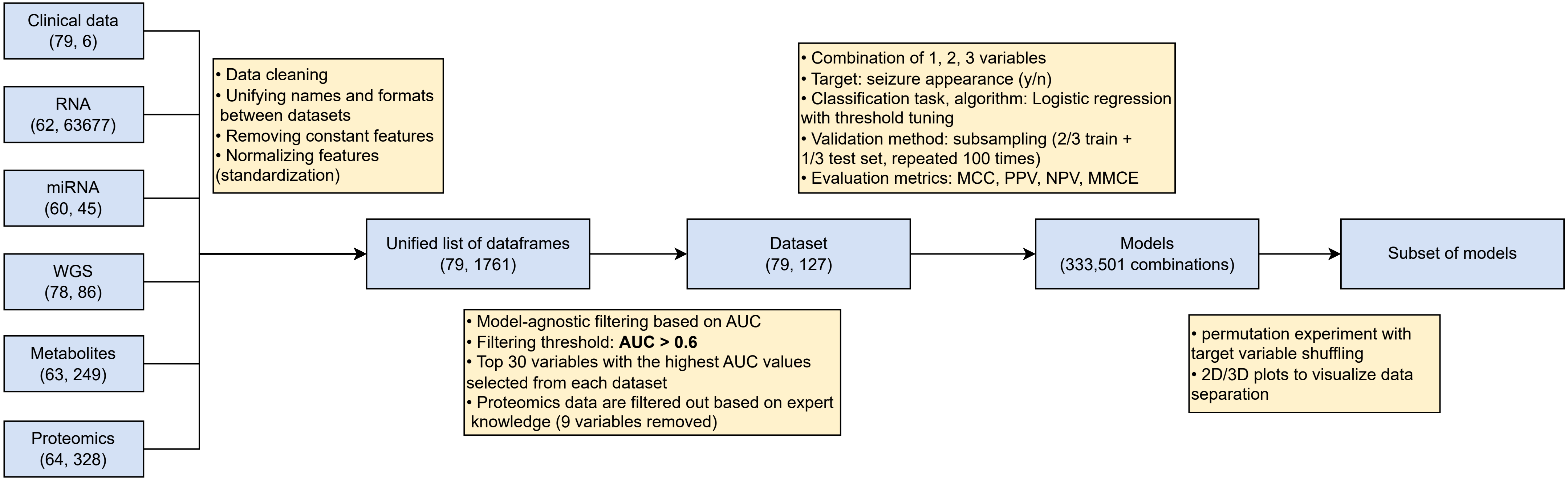

Supplementary Figure 9. Processing pipeline for classifier analysis. Blue boxes represent data flow, yellow boxes describe processing steps.
