# Supplementary material for: Molecular EPISTOP, a comprehensive multi-omic analysis of blood from Tuberous Sclerosis Complex infants age birth to two years
Source: Nat Commun. 2023 Nov 23;14:7664. doi: 10.1038/s41467-023-42855-6 (PMC10667269; doi:10.1038/s41467-023-42855-6)
Supplement: Supplementary file 3 — Description of Additional Supplementary Files [file 41467_2023_42855_MOESM3_ESM.pdf]

Description of Additional Supplementary Files

Supplementary Data 1. Clinical information and annotations for entire cohort.

Supplementary Data 2. Metabolites affected by VGB treatment. Analytes showing a statistically significant difference between individuals who received VGB vs. those that did not (age > 40 weeks; nVGB = 72; nNoVGB = 28; Wilcoxon rank sum test with continuity correction or Wilcoxon rank sum exact test; two-tailed; p-value correction using Benjamini-Hochberg prcedure).

Supplementary Data 3. Proteomic, metabolite, and RNA-Seq analytes showing a significant difference according to age. Samples were grouped into age bins (0-10 weeks, 11-40 weeks, >40 weeks of age) and tested for differences using the Kruskal-Wallis test (FDR < 0.05).

Supplementary Data 4. Pathway enrichment analyses for protein group and RNA species clusters with similar patterns during development (corresponds to Supplementary Figure 3). Samples were grouped into age bins (0-10 weeks, 11-40 weeks, >40 weeks of age). Analytes with similar patterns of change with age were clustered into 6 and 8 clusters for protein groups and RNAs, respectively. Pathway enrichment analysis was performed in R using the packages biomaRt, ReactomePA and clusterProfiler using a one sided Fisher's exact test in combination with Benjamini-Hochberg procedure for p-value adjustment (significance threshold: adjusted p-value < 0.05).

Supplementary Data 5. Analytes with differences between TSC samples without prior treatment and no seizure history (including samples that showed abnormal EEGs at sample draw), and non-TSC controls. The two-tailed Wilcoxon rank sum test was used to identify significant differences (FDR < 0.05, fold change > 1.5; p-value correction using Benjamini-Hochberg prcedure). Data was previously corrected for batch, VGB treatment (metabolite data) via Z-score correction method and age (protein, metabolite and RNAseq data) using a linear mixed model correction.

Supplementary Data 6. Pathway enrichment analysis of genes whose expression was significantly different in TSC (no history of VGB treatment nor seizure appearance) vs. age matched non TSC control samples following a one sided Fisher's exact test in combination with Benjamini-Hochberg procedure for p-value adjustment (significance threshold: adjusted p-value < 0.05).

Supplementary Data 7. Analytes that were significantly different among three groups: (group 1) non-TSC control; (group 2) TSC subjects who never developed epilepsy during the

two year course of the study, with or without vigabatrin; and (group 3) TSC subjects who did develop epilepsy (Kruskal-Wallis rank sum test and Dunn's Multiple Comparison Test (FDR < 0.05 using Benjamini-Hochberg correction for multiple hypothesis testing; fold change > 1.5). Both mixed model, and Z-score methods of correction were used (1st and 2nd sheets).

Supplementary Data 8. Pathway analysis for protein groups and RNA species that were significantly different between the TSC epilepsy group vs. controls following a one sided Fisher's exact test in combination with Benjamini-Hochberg procedure for p-value adjustment (significance threshold: adjusted p-value < 0.05).

Supplementary Data 9. The results of multi-omics predictive modeling analysis.
A. Top 100 classifiers identified as statistically significant using a permutation test, and their component analytes. The p-value was determined through a one-sided permutation test.
B. Statistics for all molecules contributing to 100 predictive models selected in classification experiment.

Supplementary Data 10. List of miRNAs analyzed from serum samples.

Supplementary Data 11. List of single nucleotide polymorphisms (SNPs) associated with epilepsy in prior studies, selected for classifier analysis.
