# Supplementary material for: Molecular EPISTOP, a comprehensive multi-omic analysis of blood from Tuberous Sclerosis Complex infants age birth to two years
Source: Nat Commun. 2023 Nov 23;14:7664. doi: 10.1038/s41467-023-42855-6 (PMC10667269; doi:10.1038/s41467-023-42855-6)
Supplement: Supplementary file 15 — Reporting Summary [file 41467_2023_42855_MOESM15_ESM.pdf]

nature portfolio

Corresponding author(s): Kwiatkowski, David

Last updated by author(s): Oct 3, 2023

Reporting Summary

Nature Portfolio wishes to improve the reproducibility of the work that we publish. This form provides structure for consistency and transparency in reporting. For further information on Nature Portfolio policies, see our Editorial Policies and the Editorial Policy Checklist.

Statistics

For all statistical analyses, confirm that the following items are present in the figure legend, table legend, main text, or Methods section.

n/a	Confirmed	
☐	☒	The exact sample size (*n*) for each experimental group/condition, given as a discrete number and unit of measurement
☐	☒	A statement on whether measurements were taken from distinct samples or whether the same sample was measured repeatedly
☐	☒	The statistical test(s) used AND whether they are one- or two-sided<br>*Only common tests should be described solely by name; describe more complex techniques in the Methods section.*
☐	☒	A description of all covariates tested
☐	☒	A description of any assumptions or corrections, such as tests of normality and adjustment for multiple comparisons
☐	☒	A full description of the statistical parameters including central tendency (e.g. means) or other basic estimates (e.g. regression coefficient) AND variation (e.g. standard deviation) or associated estimates of uncertainty (e.g. confidence intervals)
☐	☒	For null hypothesis testing, the test statistic (e.g. *F*, *t*, *r*) with confidence intervals, effect sizes, degrees of freedom and *P* value noted<br>*Give P values as exact values whenever suitable.*
☒	☐	For Bayesian analysis, information on the choice of priors and Markov chain Monte Carlo settings
☐	☒	For hierarchical and complex designs, identification of the appropriate level for tests and full reporting of outcomes
☐	☒	Estimates of effect sizes (e.g. Cohen's *d*, Pearson's *r*), indicating how they were calculated

Our web collection on statistics for biologists contains articles on many of the points above.

Software and code

Policy information about availability of computer code

Data collection	An eCRF for this study was created by Transition Technologies Science, Warsaw, Poland. Information about the eCRF is available at https://ecrf.com/en/.
Data analysis	Excel; Perseus (1.6.14.0); MaxQuant (v1.6.1.0); MultiQuant (v3.0); tophat (v.2.0.14); R (v4.1.0) – R packages used are mentioned in Materials and Methods. The complete code for processing of these data tables can be accessed via Github repository (https://github.com/JagGlo/molecular_EPISTOP).

For manuscripts utilizing custom algorithms or software that are central to the research but not yet described in published literature, software must be made available to editors and reviewers. We strongly encourage code deposition in a community repository (e.g. GitHub). See the Nature Portfolio guidelines for submitting code & software for further information.

Data

Policy information about availability of data

All manuscripts must include a data availability statement. This statement should provide the following information, where applicable:
- Accession codes, unique identifiers, or web links for publicly available datasets
- A description of any restrictions on data availability
- For clinical datasets or third party data, please ensure that the statement adheres to our policy

The raw RNA-Seq data generated for this study are held at the European Genome-phenome Archive (EGA) under the accession number: EGAS00001007264. Access to this data is controlled by a data access committee. Please email JDM at james.mills@ucl.ac.uk or DJK at dk@rics.bwh.harvard.edu for further information, reply

will occur within 24 hours. All other large data files have been combined into supplemental Tables S1A and S1B, which has been placed in our github repository along with the code for doing this analysis (https://github.com/JagGlo/molecular_EPISTOP), which has DOI: 10.5281/zenodo.8389826 [53].

Research involving human participants, their data, or biological material

Policy information about studies with human participants or human data. See also policy information about sex, gender (identity/presentation), and sexual orientation and race, ethnicity and racism.

Reporting on sex and gender	Sex was determined based on self-reporting, as well as assessment by the treating physician. Sex-based analyses were performed, and are reported in the manuscript.
Reporting on race, ethnicity, or other socially relevant groupings	Please see Table 51 and reference 8 (Kotulska K, et al. Prevention of Epilepsy in Infants with Tuberous Sclerosis Complex in the EPISTOPT rial. Ann Neurol 89, 304-314 (2021)). Race and ethnicity were not recorded for participants. All subjects were derived from European cities and one in Australia, so that the vast majority of subjects were of European ancestry.
Population characteristics	Please see Table 51 and reference 8 (Kotulska K, et al. Prevention of Epilepsy in Infants with Tuberous Sclerosis Complex in the EPISTOPT rial. Ann Neurol 89, 304-314 (2021)).
Recruitment	Infants with a diagnosis ofTSC were recruited to this study by their pediatric neurologist care providers.
Ethics oversight	The study protocol was approved by the ethical review boards of each institution at which subjects were enrolled. Please see reference 8 - Kotulska K, et al. Prevention of Epilepsy in Infants with Tuberous Sclerosis Complex in the EPISTOPT rial. Ann Neurol 89, 304-314 (2021).

Note that full information on the approval of the study protocol must also be provided in the manuscript.

Field-specific reporting

Please select the one below that is the best fit for your research. If you are not sure, read the appropriate sections before making your selection.

[x] Life sciences   [ ] Behavioural & social sciences   [ ] Ecological, evolutionary & environmental sciences

For a reference copy of the document with all sections, see nature.com/documents/nr-reporting-summary-flat.pdf

Life sciences study design

All studies must disclose on these points even when the disclosure is negative.

Sample size	A sample size of 100 TSC infant subjects was chosen initially as being a size that could be recruited at these insitutions over 5 years.
Data exclusions	Subjects were excluded if they did not meet diagnostic criteria for TSC. Data were excluded only when they did not meet pre-set quality control measures, e.g. insufficient reads from an RNA-Seq analysis.
Replication	Replication was not possible due to limited subjects, and limited samples available.
Randomization	This is described in detail in ref 8. Kotulska K, et al. Prevention of Epilepsy in Infants with Tuberous Sclerosis Complex in the EPISTOPT rial. Ann Neurol 89, 304-314 (2021). We had intended to perform a randomized controlled trial (RCT) at all 10 enrollment sites. However, the RCT was not approved by ethics boards at 4 sites, and hence subjects at those sites were enrolled in a parallel open-label trial (OLT), with treatment according to local clinical practice: preventive treatment at 2 sites and conventional treatment at 2 others. The analysis reported here was performed on the pooled population of all subjects, and hence this randomization was not relevant to this study.
Blinding	Blinding was not possible for the clinicians involved in this study. The primary sample collection and processing was performed blinded to clinical status and treatment. The data analysis reported here included grouping data according to seizure and treatment status, so it was not possible to do this in a blinded manner.

Reporting for specific materials, systems and methods

We require information from authors about some types of materials, experimental systems and methods used in many studies. Here, indicate whether each material, system or method listed is relevant to your study. If you are not sure if a list item applies to your research, read the appropriate section before selecting a response.

Materials & experimental systems

n/a	Involved in the study
☒	☐ Antibodies
☒	☐ Eukaryotic cell lines
☒	☐ Palaeontology and archaeology
☒	☐ Animals and other organisms
☐	☒ Clinical data
☒	☐ Dual use research of concern
☒	☐ Plants

Methods

n/a	Involved in the study
☒	☐ ChIP-seq
☒	☐ Flow cytometry
☒	☐ MRI-based neuroimaging

Clinical data

Policy information about clinical studies

All manuscripts should comply with the ICMJEguidelines for publication of clinical research and a completedCONSORT checklist must be included with all submissions.

Clinical trial registration	NCT02098759
Study protocol	see ref 8. Kotulska K, et al. Prevention of Epilepsy in Infants with Tuberous Sclerosis Complex in the EPISTOP Trial. Ann Neural 89, 304-314 (2021).
Data collection	Blood samples were collected at subject visits at their local hospitals for all 9 sites in Europe and 1 site in Australia. The EPISTOPtr ial was carried out from March 2014 to October 2018.
Outcomes	The primary outcome measure was the occurrence of epilepsy. Secondary outcome measures are the subject of this manuscript, identification of molecular predictors of epilepsy in this cohort.
